# Infections requiring hospitalization in the abatacept clinical development program: an epidemiological assessment

**DOI:** 10.1186/ar2984

**Published:** 2010-04-14

**Authors:** Teresa A Simon, Johan Askling, Diane Lacaille, Jarrod Franklin, Frederick Wolfe, Allison Covucci, Samy Suissa, Marc C Hochberg

**Affiliations:** 1 Global Health Economics and Outcomes Research, Bristol-Myers Squibb, Route 206 and Province Line Roads, Lawrenceville, NJ 08540, USA; 2 Department of Medicine, Clinical Epidemiology Unit, Karolinska University Hospital Solna, Rheumatology Unit d2:01, Karolinska University Hospital Solna, 171 76 Stockholm, Sweden; 3 Division of Rheumatology, Department of Medicine, Arthritis Research Centre of Canada, University of British Columbia, 895 West 10th Ave., Vancouver, BC V5Z 1L7, Canada; 4 Arc Epidemiology Unit, School of Medicine, University of Manchester, Stopford Building, Oxford Road, Manchester, M13 9PT, UK; 5 Current address: Medical School, University of Sheffield, Beech Hill Road, Sheffield, S10 2RX, UK; 6 Department of Internal Medicine, National Data Bank for Rheumatic Diseases, Arthritis Research Foundation and University of Kansas, 1035 N. Emporia, Suite 288, Wichita, KS 67214, USA; 7 Global Biostatistics, 311 Pennington Rocky Hill Road, Bristol-Myers Squibb, Hopewell, NJ 08525, USA; 8 Center for Clinical Epidemiology, Lady Davis Research Institute, Jewish General Hospital, 3755 Cote Ste-Catherine, Montreal, Québec H3T 1E2, Canada; 9 Departments of Medicine and Epidemiology and Preventive Medicine, University of Maryland School of Medicine, 10 S. Pine St., MSTF 8-34, Baltimore, MD 21201, USA

## Abstract

**Introduction:**

Patients with rheumatoid arthritis (RA) have an increased risk of infection and this risk appears to be higher with anti-TNF (tumor necrosis factor) agents. We pooled data from the cumulative abatacept RA clinical development program, both double-blind and open-label periods, to estimate the incidence rates (IRs) of infections requiring hospitalization including pneumonia and opportunistic infections, in comparison with RA patients treated with non-biologic disease-modifying antirheumatic drugs (DMARDs) from several reference cohorts.

**Methods:**

Infections reported in seven abatacept clinical trials of RA patients (double-blind and open-label periods) were tabulated. Comparisons were made between the observed IRs in abatacept-treated patients and those in over 133,000 patients exposed to non-biologic DMARDs in six reference RA cohorts. Age- and sex-adjusted IRs of infections requiring hospitalization, including pneumonia (most frequent hospital infection), were used to estimate the expected IRs with abatacept by the method of indirect adjustment. Standardized incidence ratios (SIR) and 95% CI were calculated comparing incidence in the cumulative abatacept experience with incidence in each RA cohort.

**Results:**

A total of 1,955 (double-blind period) and 4,134 (double-blind + open-label periods with a cumulative exposure of 8,392 person-years) abatacept-treated RA patients were analyzed. Observed IRs for infections requiring hospitalization during the double-blind period were 3.05 per 100-patient years for abatacept-treated patients and 2.15 per 100 patient years for placebo. In the cumulative population, observed IR for infections requiring hospitalization was 2.72 per 100-patient years. Rates for abatacept were similar to expected IRs based on other RA non-biologic DMARD cohorts.

**Conclusions:**

IRs of infections requiring hospitalization and pneumonia in abatacept trials are consistent with expected IRs based on reference RA DMARD cohorts. RA patients are at higher risk of infection compared with the general population, making the RA DMARD cohorts an appropriate reference group. The safety of abatacept, including incidence of infections requiring hospitalization, will continue to be monitored in a post-marketing surveillance program.

## Introduction

Patients with rheumatoid arthritis (RA) have been shown to have an increased risk of infection compared with the general population [1,2]. Some studies have also shown that this risk varies according to treatment of RA patients, with a higher risk of infections with anti-TNF (tumor necrosis factor) agents compared with non-biologic disease-modifying antirheumatic drug (DMARDs) [3,4]. Treatment with biologic agents is generally a highly effective approach for patients with RA, but may compromise host defense mechanisms involved in protection from infections and tumor surveillance; adverse events, serious infections in particular, are therefore a concern [4].

Abatacept is the first in a class of agents for the treatment of rheumatoid arthritis (RA) that selectively modulates the CD80/CD86:CD28 co-stimulatory signal required for T-cell activation [5]. Abatacept has demonstrated efficacy in the treatment of rheumatoid arthritis (RA) [6-11]. While the safety and tolerability of abatacept has been described in the individual randomized trials [12], it is prudent to evaluate the overall risk of infections requiring hospitalization (hospitalized infections), of hospitalized pneumonia, and of tuberculosis (TB) and other opportunistic infections in the cumulative trial experience.

To date, aggregate double-blind infection rates (serious and those requiring hospitalization) following abatacept treatment have been published in abstract form only and limited data have been published on the longer-term cumulative incidence from the integrated (double-blind and open-label) data of all abatacept exposed patients [13,14]. Overall, a serious infection is an infection that results in death, requires or prolongs a hospitalization, is life-threatening or deemed as medically important by the trial investigator. Serious infection incidence rates from the integrated randomized double-blind, placebo-controlled trials (RCTs) of abatacept [6-11] were 3.47/100 patient-years (py) and 2.41/100 py for abatacept and placebo, respectively [13]. Similarly, the incidence rates of infections requiring hospitalization (a subset of serious infections) in the combined double-blind placebo-controlled trials was 3.05/100 py and 2.16/100 py for abatacept and placebo, respectively [14].

In this paper, we report on infections requiring hospitalizations in the cumulative experience with abatacept from RCTs, including both the double-blind and the open-label phases. Since no control groups are available for the open-label extension phases, we have used external RA cohorts to serve as comparator groups so that the rates observed with abatacept are placed into context with comparable, real-world RA populations treated with DMARDs. This permitted the evaluation of infection risk over longer periods than the shorter follow-up of RCTs, and allowed us to combine the experience from multiple trials.

## Materials and methods

All person-time from all patients exposed to abatacept in the clinical development program (CDP) were included for the computation of infections requiring hospitalization (hospitalized infections), pneumonia requiring hospitalization (hospitalized pneumonia), and TB incidence rates. Several large population-based registries were utilized to establish a range of reference hospitalized infection incidence rates in RA patients treated with non-biologic DMARDs. These were compared with the incidence rates of infections that lead to hospitalization in abatacept-treated patients. The method of indirect comparison was applied. Data reflect all patients in clinical trials treated with abatacept through December 2006. Expected events in the RA cohorts are adjusted for age and gender and account for exposure.

### Study design

This was both a comprehensive pooled analysis of trial data, and an observational epidemiological study examining hospitalized infections, hospitalized pneumonia, and infections of interest (specifically TB), based on the comparison between the occurrence of infections requiring hospitalization in all patients ever exposed to abatacept in the CDP with the occurrence of these infections requiring hospitalization in six observational cohorts of RA patients in Europe and North America. Data reflect all patients in the abatacept CDP, including double-blind (DB) and open-label (OL) phases of RCTs, through December 2006.

### Data sources

Clinical safety data from seven abatacept RA clinical trials were included in the analyses [6-11,15,16]. Table 1 presents these studies. Exclusion criteria and TB screening were consistent across all trials except for abatacept researched in rheumatoid arthritis patients with an inadequate anti-TNF response to validate effectiveness (ARRIVE) where there were 23 patients who were purified protein derivative (PPD) positive. Exclusions for TB and serious infections included active TB requiring treatment within the previous three years, PPD-positive subjects who had not received adequate chemoprophylaxis or prior Bacillus Calmette-Guerin (BCG) immunization, and subjects with any serious bacterial infection (such as pneumonia, renal infection, or sinusitis), unless treated and resolved with antibiotics, or chronic bacterial infection (such as pyelonephritis and chest infection with bronchiectasis) in the previous three months. Of note, protocol IM101-031 enrolled RA patients with comorbid conditions including diabetes and chronic obstructive pulmonary disorder (COPD).

**Table 1:** Description of the abatacept clinical trials included in the current analysis

Study name	Study design/study title/DB enrollment period	Duration of double-blind period (months)	abatacept	PBO	Open-label extension
IM101101 [6] Phase IIB	Randomized, placebo-controlled, double-blind/2001 to 2002	12	85	36	80
IM101100 [7,8] Phase IIB	Randomized, dose-ranging, placebo-controlled, double-blind/2001 to 2002	12	220	119	219
ATTAIN [9] IM101029 Phase III	Randomized, placebo-controlled, double-blind/Abatacept Trial in Treatment of Anti-TNF INadequate responders/2002 to 2003	6	258	133	317
AIM [10] IM101102 Phase III	Randomized, placebo controlled, double-blind/Abatacept in Inadequate responders to MTX/2002 to 2003	12	433	219	539
ASSURE [11] IM101031 Phase III	Randomized, placebo-controlled, double-blind/Abatacept Study of Safety in Use with other RA therapies/2002 to 2003	12	959	482	1184
	Total double-blind 5 core above		1955	989	2689**
ATTEST [15] IM101043	Abatacept or infliximab versus placebo, a Trial for Tolerability, Efficacy and Safety in Treating RA/2005 to 2006	12	156	110	236* (132 aba, 104 placebo, 136 infliximab)
ARRIVE [16] IM101064	Abatacept Researched in Rheumatoid arthritis patients with an Inadequate anti-TNF response to Validate Effectiveness/2005 to 2006	6 (open-label)	1046		530

*IM101043, without infliximab arm; **Number represents total number of abatacept exposed patients exposed during both double-blind and open-label; five core trials N = 2,689, overall N = 4,134.

The observational RA comparison groups used to perform the indirect comparison analyses were derived from the following: the British Columbia (BC) population-based RA Cohort in Canada, the Norfolk Arthritis Register (NOAR) in the UK, the National Data Bank for Rheumatic Diseases (NDB) in the USA, the Early Rheumatoid Arthritis Register and the Swedish Inpatient Hospitalization in Sweden (Sweden ERA, Sweden INPT), and the PharMetrics database in the US. Characteristics of these data sources have been previously described in the literature [2,17-20]. The six observational cohorts were selected for their ability to provide the patient population of interest (RA patients receiving non-biologic DMARD only), age- and sex-specific incidence rates (IRs) of the specified outcomes, and the ability of the investigators to complete the analyses for regulatory filings. Table 2 presents the characteristics of these databases with respect to cohort characteristics (type and number of patients), time period covered, and data availability.

**Table 2:** Characteristics of RA data sources used for epidemiologic analysis.

Data source	BC	PharMetrics	NOAR	NDB	Swedish ERA	Swedish inpatient*
Country	Canada	United States	United Kingdom	United States	Sweden	Sweden
Data type	Administrative data on physician visits, hospitalizations and medications	Administrative Claims data	Patient Questionnaire & assessment	Patient Questionnaire	Electronic medical records, patient assessment	Medical Records
Time covered	1996 to 2002	1998 to 2002	1990 to 1999	1998 to 2003	1994 to 2003	1990 to 2003
Number of RA patients in cohort	12,337	24,530	523	10,499	3,703	53,067
DMARD users	Prevalent users	Prevalent users	Incident users	Prevalent users	Incident users	Prevalent users
Outcome (infection) ascertainment	ICD-9 codes on claims and discharge summaries	ICD-9 codes on claims	ICD-9 codes in linked medical records	Patient-reported and verified by medical and hospital records	ICD-10 codes and verified by linking to hospital registry	ICD-10 codes in hospital registry

*The Sweden inpatient cohort is not a DMARD-only group and may contain patients on biologic therapy.

Characteristics of data sources used for identification of RA patients included in the epidemiologic analysis. BC: British Columbia RA Cohort; NOAR: Norfolk Arthritis Register; NDB: National Data Bank for Rheumatic Diseases; Sweden ERA: Sweden Early Rheumatoid Arthritis Register.

The PharMetrics analyses were conducted on data held by the sponsor of the current study, while the data sources used for the other cohort analyses are proprietary and reside with the affiliated university or research center.

For this study, each registry and data source holder obtained ethics or Institutional Review Board (IRB) approval in accordance with local requirements. Approvals are maintained and updated regularly as required by local law for each individual study.

### Exposure

The cumulative integrated abatacept experience included 4,134 abatacept-treated patients representing 8,392 person-years of abatacept exposure from seven clinical trials (Table 1). Because 80% of subjects in the abatacept CDP were on background non-biologic DMARD therapy during the trials (most frequently methotrexate), and almost all had prior exposure to non-biologic DMARDs, the most relevant reference group for comparison was considered to be non-biologic DMARD-treated patients because they would be similar to the placebo patients. Therefore, non-biologic DMARD patients were retrospectively identified from each observational data source and became part of the study cohort. Patients with a known exposure to a biologic agent were excluded from these observational database cohorts. In total, approximately 137,000 non-biologic DMARD-treated patients with RA from the observational cohorts were identified and included in the analyses.

### Study subjects

Patients included all those who were ever exposed to abatacept treatment anytime during the DB and OL periods of the cumulative abatacept CDP (Table 3). Subjects who agreed to enter the OL period after completing the DB period were enrolled; no specific response criteria or additional screening was required (Table 1).

**Table 3:** Baseline demographics and clinical characteristics of abatacept clinical trial patients and RA DMARD cohorts

	Abatacept CDP (N = 4,134)	BC (N = 12,337)	NDB (N = 10,499)	PharMetrics (N = 52,444)	NOAR (N = 523)	Sweden ERA (N = 3,703)	Sweden Inpatient (N = 53,067)
Age, n (%)							
18 to 44	1,015 (25)	3,088 (25)	1,442 (14)	15,733 (30)	109 (21)	782 (21)	4,776 (9)
45 to 74	2,988 (72)	7,840 (64)	7,586 (73)	35,137 (67)	366 (70)	2,421 (66)	29,718 (56)
≥ 75	131 (3)	1,409 (11)	1,438 (14)	1573 (3)	48 (9)	500 (14)	18,573 (35)
Female, n (%)	3,323 (80)	8,936 (72)	7,971 (76)	18,569 (76)	357 (68)	2,589 (70)	37,678 (71)
Duration of RA, n (%)							
<5 years	1,353 (33)	4,890 (40)	2,726 (29)*	NA	523	3,703	NA
5 to 10 years	1,192 (29)	4,206 (34)	1,902 (20)*	NA	0	0	NA
>10 years	1,586 (38)	3,241 (26)	4,716 (50)*	NA	0	0	NA
Concomitant medications, n (%)^†^							
Oral corticosteroids	2,657 (64)	8,121 (66)	4,588 (44)	11,504 (48)	194 (37)	NA	NA
NSAIDS	3,113 (75)	11,001(89)	6,820 (65)	NA	416 (80)	NA	NA
Total follow-up (years)							
Mean	2.1	4.9	3.3	2.2	7.9	3.6	5.6
Median	1.8	6.0	2.5	2.0	9.3	NA	NA

*RA duration was not collected for every subject in the NDB; therefore, n = 9,344 for this variable. ^†^Use of concomitant medications at baseline is presented for the abatacept trial population, whereas, use during follow-up is presented for the RA cohorts (where available). BC, British Columbia population-based RA Cohort; NDB, National Data Bank for Rheumatic Diseases; NOAR, Norfolk Arthritis Register; Sweden ERA, Sweden Early Rheumatoid Arthritis Register. NA = not available.

The RA DMARD cohorts examined in this study were multinational, had varying durations of follow up, and used different case ascertainment methods (Table 2). Data sources consisted of claims, questionnaires and assessments, collected over a period spanning 14 years, between 1990 and 2003. The cohort populations varied from around 500 patients to over 53,000. Women constituted more than two-thirds of each cohort.

Across all cohorts, most patients (63% to 72%) were between 45 and 74 years of age. While older age groups were underrepresented in the abatacept CDP, comparisons of infections requiring hospitalization rates were adjusted for age. Duration of RA was not available for the PharMetrics and Swedish inpatient cohorts. The duration of RA for the abatacept patients was most similar to the BC and NDB cohorts; whereas the early RA cohorts followed patients from disease onset. These cohorts represent a range in RA disease duration.

Mean patient-years of follow-up per subject across RA DMARD cohorts ranged from 2.2 to 7.9 py. Across all cohorts in which RA medication use data were collected, in addition to non-biologic DMARDs, use of glucocorticoids ranged from 37% (NOAR) to 66% (BC), while use of non-steroidal anti-inflammatory drugs (NSAIDs) ranged from 65% (NDB) to 89% (BC). The demographic characteristics of the RA patients are presented by cohort in Table 3.

### Outcome (infection) ascertainment

Pre-specified outcomes included overall infections requiring hospitalization pneumonia requiring hospitalization (the most frequently reported infection requiring hospitalization) and TB. Infections in this study were identified by international classification of diseases (ICD) -9 and ICD-10 diagnostic codes in the BC, NOAR, PharMetrics, Swedish Inpatient, and the Sweden ERA cohorts. Specifically, in the BC and PharMetrics cohorts, hospitalized infections were identified from the ICD-9 diagnostic codes recorded on discharge summaries of hospitalization data. For the NOAR, hospitalized infection information was obtained by linkage of the cohort with the electronic records system of the region's only major hospital. In the Sweden ERA cohort, information on hospitalized infections was acquired through linkage to the inpatient hospitalized discharge diagnosis and hospital discharge diagnoses were used for the Swedish Inpatient data source records. In the NDB, hospitalized infections were identified from semi-annual questionnaires sent to participants. All reports of hospitalized infections were validated with hospital and medical records. For patients in the abatacept CDP, hospitalized infections were identified from all adverse event (AE) reports and validated through special event forms; events were included regardless of relationship to study drug.

### Analyses

Baseline demographic and clinical characteristics were summarized using descriptive statistics for continuous or categorical variables as appropriate. In the abatacept CDP, all episodes of hospitalized infections, hospitalized pneumonia and TB cases were counted from the start of therapy until the first event or end of treatment period + 56 days, whichever occurred first.

Rates were computed for the double-blind period, as well as the cumulative (double blind and open label) study period. In the RA DMARD observational cohorts, person-time and incidence of hospitalized infections, hospitalized pneumonia and TB were calculated from the first recorded non-biologic DMARD exposure until the first event or the end of follow-up, whichever occurred first. The IRs for each outcome of interest in the RA DMARD cohorts were standardized to the age (10-year interval) and sex distribution of the abatacept clinical trial experience by the method of indirect standardization. All cases of TB are reported. TB rates were not standardized due to the insufficient number of cases therefore overall rates from each data source are presented. For all outcomes, an indirect comparison was computed using the number of events observed in the abatacept CDP and the number of events expected given the same age, sex, and exposure distribution in the RA cohorts.

To estimate the relative risk (RR) of hospitalized infections and pneumonia in the abatacept CDP relative to that in each of the six RA DMARD cohorts, standardized incidence ratios (SIRs) were calculated by an indirect comparison method of dividing the observed numbers of infections in the abatacept CDP by the expected numbers from the RA DMARD cohorts. The expected numbers were calculated by multiplying the hospitalized infection rates in each of the six RA cohorts by the observed person-years at risk, stratified by sex and the 10-year age group. Rate ratios calculated between groups based on incidence rates of events was calculated by the method of DerSimonian and Laird [21]. Furthermore, we computed a summary SIR estimate (and 95% CI) combining the SIRs from the six DMARD cohorts based on the meta-analysis method of DerSimonian and Laird [21]. This method uses a random effects model which considers both within-study and between-study variation by incorporating the heterogeneity of effects in the overall analysis. For all SIRs, 95% CI was calculated using the Wilson and Hilferty approximation [22]. Statistical analyses were performed using the SAS software package (SAS Institute, Cary, North Carolina, USA). Crude incidence rates from the RA DMARD cohorts were reported for TB and compared with the crude incidence rate in the abatacept CDP.

## Results

The cumulative integrated abatacept experience included 4,134 abatacept-treated patients representing 8,392 person-years of abatacept exposure from seven clinical trials (Table 1). Because 80% of subjects in the abatacept CDP were on background non-biologic DMARD therapy during the trials (most frequently methotrexate), and almost all had prior exposure to non-biologic DMARDs, the most relevant reference group for comparison was considered to be non-biologic DMARD-treated patients because they would be similar to the placebo patients

Presented in Table 4 are the total number of events as well as the incidence rates for infections requiring hospitalization (hospitalized infections) and pneumonia requiring hospitalization (hospitalized pneumonia) in the cumulative abatacept CDP (observed) and RA cohorts (expected). The incidence rate for hospitalized infections in the DB periods of the RCTs was 3.05/100 py for abatacept and 2.15/100 py for placebo (rate ratio 1.42; 95% CI: 0.82 to 2.45).

**Table 4:** Observed and expected IRs of hospitalized infections, and pneumonia, in abatacept CDP and RA cohorts.

Data source	Counts	Hospitalized infections* IR**/100 py (95% CI)	Counts	Hospitalized pneumonia* IR**/100 py (95% CI)
Observed (Double-blind trial data)				
Abatacept Population N = 1,955	51	3.05 (2.3, 4.0)	12	0.71 (0.4, 1.3)
Placebo Population N = 989	17	2.15 (1.26, 3.45)	4	0.50 (0.14, 1.29)
Observed (Long-term open-label trial data)				
Cumulative Abatacept Trial Population (DB + OL) N = 4,134	221	2.72 (2.37,3.10)	54	0.65 (0.47, 0.82)
Expected (Observational cohort data)				
BC	252	3.00 (2.65, 3.40)	66	0.79 (0.62, 1.01)
NDB	NA	NA	110	1.31 (1.09, 1.58)
PharMetrics	296	3.53 (3.15, 3.96)	106	1.26 (1.04, 1.53)
NOAR	118	1.41 (1.18, 1.69)	22	0.27 (0.18, 0.40)
Sweden ERA	154	1.83 (1.57, 2.15)	44	0.53 (0.39, 0.71)
Sweden INPT	329	3.92 (3.52, 4.37)	87	1.04 (0.84, 1.28)

No cases of coccidioidomycosis, cryptococcus, histoplasmosis, nocardiosis, or *Pneumocystis carinii *pneumonia were observed. *Rates in external RA cohorts are age-(10-year groups) and sex-adjusted to the abatacept trial population. **Rates are cases per 100 py; ^†^crude, unadjusted rate. BC: British Columbia RA Cohort; NA: not available; NOAR: Norfolk Arthritis Register; NDB: National Data Bank for Rheumatic Diseases; Sweden ERA: Sweden Early Rheumatoid Arthritis Register

For hospitalized pneumonia, the incidence rate in the DB periods of RCTs was 0.71/100 py for abatacept and 0.50/100 py for placebo (Table 4). The incidence rate in the cumulative abatacept population for hospitalized infections was 2.72/100 py, which falls within the range of expected values calculated for the external cohorts (1.41 to 3.92/100 py) (Table 4). The incidence rate in the cumulative abatacept population for hospitalized pneumonia was 0.65/100 py, which also falls within the range of incidence rate values calculated for the external cohorts (0.27 to 1.31/100 py).

The overall incidence rate of TB in the cumulative abatacept clinical trial experience was low and comparable with the RA cohorts (Table 5). Three cases of TB were reported for a cumulative rate of 0.04/100 py. There was no increased incidence of TB compared with the RA cohorts.

**Table 5:** Observed overall incidence rates of tuberculosis in the abatacept CDP and RA cohorts

Cohort	Tuberculosis IR/100 py (95% CI)
Observed IR	
DB Abatacept Population	0.06 (0.0 to 5.6)
DB Placebo Population	0.13 (0.0 to 5.6)
Cumulative Abatacept Population (DB + OL)**	0.04 (0.01 to 0.10)
Expected IR	
BC	0.03 (0.01 to 0.05)
NDB	0.02 (0.01 to 0.04)
PharMetrics	0.02 (0.01 to 0.04)
NOAR	0.013*
Sweden ERA	0.026 (0.003 to 0.094)
Sweden INPT	0.05 (0.04 to 0.06)

*95% CI not available. **n = 3.

Overall, opportunistic infections and TB were rare in the abatacept CDP. Other opportunistic infections reported included one event each of aspergillosis, blastomycosis, and systemic Candida infection reported. No events resulted in hospitalization. No cases of coccidioidomycosis, cryptococcus, histoplasmosis, nocardiosis, or *Pneumocystis carinii *pneumonia were observed.

**Figure 1:**
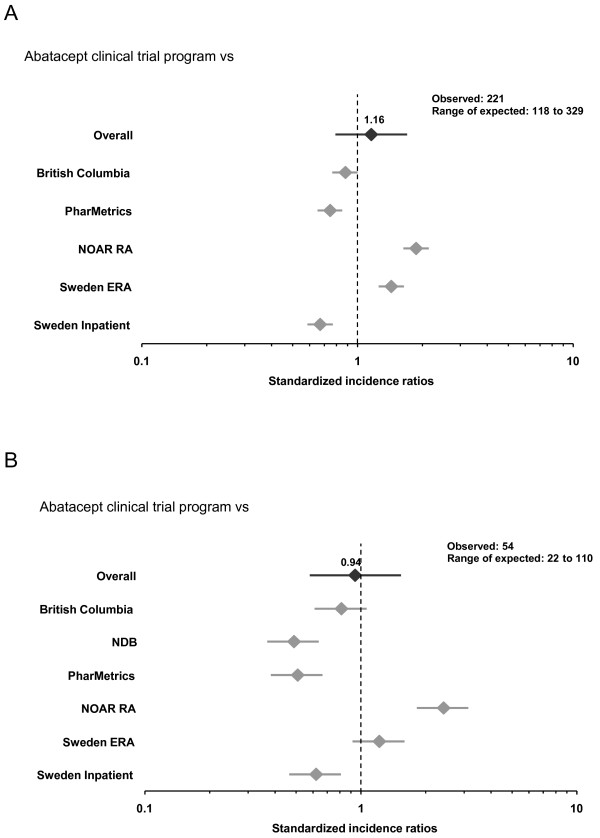
**Standardized incidence ratios (SIR) comparing the risk of hospitalized infections (A) and pneumonia (B) in abatacept CDP and RA cohorts**. Expected events are age-adjusted (10-year age groups) and gender-adjusted and account for exposure. Data reflect all patients in clinical trials treated with abatacept through December 2006. Overall SIRs were derived through a meta-analysis of SIRs using individual cohorts.

Standardized incidence ratios (SIRs) of hospitalized infections and pneumonia are listed according to data source (Figure 1A and 1B, respectively). These SIRs represent the risk of infections requiring hospitalization in the cumulative abatacept experience compared with each of the RA cohorts. Using the DerSimonian and Laird [21] method, a pooled SIR for hospitalized infections and hospitalized pneumonia were calculated. The pooled SIR for hospitalized infection was 1.16 (95% CI 0.79 to 1.70) and 0.94 (95% CI 0.58 to 1.53) for hospitalized pneumonia.

## Discussion

As placebo is the best comparator but is generally limited to use within the double-blind phase of an RCT, this study provides context for infections requiring hospitalization in patients with an inadequate response to methotrexate or an anti-TNF agent who were treated with long-term exposure to abatacept in the clinical development program.

Bongartz and Saillot has shown an increase in serious infections in the double-blind phase of biologic studies when compared to placebo [3,23]. Previously stated, results from the double-blind randomized trials suggest an increase in serious infections: 3.47/100 patient-years (py) and 2.41/100 py for abatacept and placebo, respectively [13] (rate ratio 1.44 (0.86 to 2.42)). This serious infection rate ratio of 1.44 (0.86 to 2.42) is within the lower limit of the 95% CI for the pooled odds ratio reported in the meta-analysis of serious infections in anti-TNF controlled trials by Bongartz (pooled Mantel-Haenszel OR 2.0 (1.3 to 3.1)) [3] and similar to the fixed combined OR of 1.35 (0.79 to 2.32) reported by Saillot [23].

The incidence rate for hospitalized infections (a subset of serious) in the DB periods of the RCTs was also increased however again not significant: 3.05/100 py for abatacept and 2.15/100 py for placebo (rate ratio 1.42; 95% CI: 0.82 to 2.45).

The cumulative data presented here suggest that the observed incidence rates of infections requiring hospitalization (hospitalized infections) hospitalized pneumonia, and TB in patients ever treated with abatacept in the cumulative CDP (both double-blind and open-label) is in the same order of magnitude as those expected from cohorts of RA patients treated with non-biologic DMARDs.

As patients who are enrolled in RCTs move from the double-blind to open label phases, the lack of a control group to monitor the occurrence of adverse events in the long term becomes challenging. It is difficult to assess whether the risk of infection in patients with RA is due to underlying RA, as suggested by Dobloug, Fox and Koetz [24-26], or by the treatments administered. Some studies have demonstrated that the use of corticosteroids may increase infection risk [19,27-29] as well as anti-TNF agents given to patients with RA [30-32]. With potential confounding by disease activity, disease severity, duration of disease, presence of comorbid conditions, and concomitant treatment, it becomes challenging to determine a true association between the risk of infections and a new treatment.

To date, there have not been any similar such studies evaluating cumulative exposure of a biologic agent to an external cohort. There have been a number of observational studies reporting rates of infection by treatments and there are cumulative and open-label study periods that have been reported. However none have offered a combined evaluation as the analysis reported here.

When conducting pharmacoepidemiologic studies, it has been recommended that at least two cohorts be evaluated to establish the extent of reproducibility [33]. The resulting variation in IRs among the cohorts provides a useful range of estimates for comparison, which is preferable to a single cohort IR. Also, published literature suggests that RA patients are at higher risk of infection compared with the general population, rendering the RA DMARD observational cohorts an appropriate reference group and not the general population [2,29].

There have been a number of published papers evaluating the risk of infections in RA patients where the exposure, outcomes, and risk measurement were not consistent across studies. These definitions include, but are not limited to, the regulatory definition of serious, hospitalized infections, and the possible inclusion of administration of IV antibiotics [23]. For transparency, the authors defined the outcomes and present the number of events, incidence rates, and percentages (proportions) for the RA cohorts and abatacept CDP.

This study has several limitations, including those inherent to its design. The data collected and analyzed from the RA cohorts were not primarily collected for this type of study. The limitations associated with the use of external control groups include, but are not limited to, differences in physician management and diagnoses of RA, the inclusion of both prevalent and new users of DMARD agents, differences in the ascertainment and verification of outcomes, length of follow-up, validity of RA diagnosis, disease duration, and severity of disease. We acknowledge that these variables may be different among the RA cohorts. Although the abatacept population appeared to be demographically similar to the cohorts, clinical trial patients are inherently different. The abatacept CDP enrolled mainly prevalent, stable non-biologic DMARD users who had an inadequate response to their current therapy; thereby implying more severe disease in these patients. The RA cohort populations were a diverse group of both prevalent and new non-biologic DMARD users. It is difficult to know how comparable the RA cohorts are to the abatacept trial population.

The RA cohort populations are potentially more stable in that for this analysis the population had to be on a non-biologic DMARD throughout follow-up without addition of a biologic therapy. However, the non biologic DMARD treatment in these groups could be altered during follow-up such that non biologic DMARD therapy could be increased, decreased, or another non-biologic treatment could be added to the current regimen all together. As with any population (trial or observational), it is difficult to know if a patient was previously exposed to a biologic agent for RA or had any other indicated comorbid condition (psoriasis, transplant) prior to entry into the trial or cohort. Trial patients may be monitored more closely for adverse events, which might overestimate our calculated SIRs. However, some of the cohorts have pre-specified observation times for patients enrolled in the registry (for example, NOAR). TB screening prior to entry in the trials may have resulted in a lower incidence of TB in the abatacept CDP. The exclusion of patients with a recent infection, suggesting that enrolled patients are potentially healthier, may have resulted in a lower overall incidence of infections, however the authors did apply this exclusion to the data to be more reflective of the trial population. We were not able to adjust for potential confounders such as severity of disease (rheumatoid factor (RF) or anticitrullinated protein/peptide antibody (ACPA) status), ethnicity, co-morbidities, and use of non-DMARD medications (for example, NSAIDs and corticosteroids) due to unavailability of data on these variables in most external cohorts. Finally, each of the databases used in this study may be associated with specific limitations, such as uncertainty surrounding diagnostic accuracy in administrative claims databases (for example, BC), small cohort size (for example, NOAR), use of self-reporting (for example, NDB), and so on. However, these individual limitations were minimized by the use of multiple cohorts, resulting in a range of references that provided relatively consistent results.

This study has several strengths. The authors predefined exposure and outcome criteria in an attempt to harmonize the methods applied to this analysis including exposure to non-biologic DMARDs, definition of events (hospitalized infections), and the computation of person-time in the evaluation of infections in the abatacept CDP and the RA cohorts.

In randomized trials, serious infections are defined as infection resulting in death, life threatening, requiring inpatient hospitalization or prolongation of a hospitalization, resulting in disability, a congenital anomaly or a medically important event based on medical and scientific judgment. This definition is difficult to apply to observational cohort data. Hence, 'infections resulting in hospitalization' was used since this outcome can be easily identified in all external observational RA data sources.

The effort to harmonize the methods used in these cohorts may seem trivial; however, Solomon and colleagues outlined the importance and complexities of defining exposure risk windows, comparators and endpoints, and when different, the challenge in interpreting data from epidemiological studies published independently [34]. The analyses presented here from six RA cohorts were done in collaboration with registry and observational data holders in an attempt to control these key methodologic issues.

Despite geographic differences and differences in ascertainment methods, the ranges of age- and sex-adjusted IRs were relatively similar among the various RA cohorts. The Early RA cohorts provided IRs on less severe patients with shorter duration of disease where NDB and BC have patients with longer disease duration. Additional strengths include the choice of comparison group. We were able to include in this study, as a reference group, only those patients treated with non-biologic DMARDs from the respective RA cohorts. Given that non-biologic DMARDs comprised the background therapy in all abatacept-treated patients in the clinical trials, this constituted an appropriate reference group for comparing risk of infections.

There have been few opportunistic infections observed throughout the abatacept CDP. TB was the most common, with a total of three events reported in 4,134 patients over a total of 8,392 person-years, none of which resulted in hospitalization.

## Conclusions

In conclusion, the comparison of data from the cumulative abatacept clinical trials and external RA cohorts exposed to non-biologic DMARDs puts the ABA double-blind trial populations and cumulative abatacept experience into perspective, and suggests that in the long-term, the risk of hospitalized infection and hospitalized pneumonia following abatacept treatment in these open-label trials is in the same order of magnitude as that of patients on non-biologic DMARD therapy. Incidence rates and number of actual events reported in the cumulative abatacept trial population fall within the range of those expected based on infection rates in external RA cohorts.

## Abbreviations

ACPA: anticitrullinated protein/peptide antibody; AE: adverse event; BC: British Columbia; BCG: Bacillus Calmette-Guerin; CDP: clinical development program; CI: confidence interval; COPD: chronic obstructive pulmonary disease; DB: double blind; DMARD: disease-modifying antirheumatic drug; ICD: international classification of diseases; IR: incidence rate; IRB: institutional review board IV: intravenous; NDB: the National Data Bank for Rheumatic Diseases in the USA; NOAR: the Norfolk Arthritis Register in the UK; NSAID: non-steroidal anti-inflammatory drug; OL: open label; PPD: purified protein derivative; PY: patient years; RA: rheumatoid arthritis; RCT: randomized controlled clinical trial; RF: rheumatoid factor; RR: relative risk; SAS: Statistical Analysis System; SIR: standardized incidence ratio; Sweden ERA: the Early Rheumatoid Arthritis Register in Sweden; Sweden INPT: the Swedish Inpatient Hospitalization; TB: tuberculosis; TNF: tumor necrosis factor.

## Competing interests

TS and AC are current full-time employees of Bristol-Myers Squibb. JA reports having been an invited speaker at meetings sponsored by Schering-Plough and Abbott. JF reports having received research funding from Bristol-Myers Squibb. DL reports receiving research funding from Bristol-Myers Squibb for the research presented in this manuscript and has participated in advisory meetings supported by Bristol-Myers Squibb. FW reports having received research grants from Bristol-Myers Squibb, Centocor, Abbott, Amgen and UCB. SS reports having served as an advisor and participating as a speaker in scientific meetings for AstraZeneca, Boehringer Ingelheim, GlaxoSmithKline, Pfizer, and Sepracor. SS also reports receiving research funding from AstraZeneca and GlaxoSmithKline. MH reports serving as a consultant to Amgen, Bristol-Myers Squibb, Abbott, UCB and Roche.

## Authors' contributions

TS conceived the study, participated in the data analyses, and prepared and revised the manuscript. JA provided data from the Swedish ERA and Inpatient cohorts, reviewed and revised the manuscript. DL provided data from the BC Cohort reviewed and revised the manuscript. JF provided data from the NOAR cohort reviewed and revised the manuscript. FW provided data from the NDB reviewed and revised the manuscript. AC reviewed and validated all computations used in the statistical analyses. SS contributed to the PharMetrics analyses, performed the SIR meta-analyses, reviewed and revised the manuscript. MH contributed to the PharMetrics analyses, provided medical input reviewed and revised the manuscript. All authors read and approved the final manuscript. Authors were not paid for their contribution to this manuscript. Their institutions did receive a grant funded by BMS to perform the analyses.

## Supplementary Material

Additional file 1 **Abatacept epidemiology study group members**. A Word file containing a complete list of all members of the Abatacept Epidemiology Study Group. Click here for file

## References

[B1] Doran MF Crowson CS Pond GR O'Fallon WM Gabriel SE Frequency of infection in patients with rheumatoid arthritis compared with controls: a population-based study Arthritis Rheum 2002 46 2287 2293 10.1002/art.10524 12355475

[B2] Smitten AL Choi HK Hochberg MC Suissa S Simon TA Testa MA Chan KA The risk of hospitalized infection in patients with rheumatoid arthritis J Rheumatol 2008 35 387 393 18260176

[B3] Bongartz T Sutton AJ Sweeting MJ Buchan I Matteson EL Montori V Anti-TNF antibody therapy in rheumatoid arthritis and the risk of serious infections and malignancies: systematic review and meta-analysis of rare harmful effects in randomized controlled trials JAMA 2006 295 2275 2285 10.1001/jama.295.19.2275 16705109

[B4] Dixon WG Symmons DP Lunt M Watson KD Hyrich KL Silman AJ Serious infection following anti-tumor necrosis factor alpha therapy in patients with rheumatoid arthritis: lessons from interpreting data from observational studies Arthritis Rheum 2007 56 2896 2904 10.1002/art.22808 17763441 PMC2435418

[B5] Tan P Anasetti C Hansen JA Melrose J Brunvand M Bradshaw J Ledbetter JA Linsley PS Induction of alloantigen-specific hyporesponsiveness in human T lymphocytes by blocking interaction of CD28 with its natural ligand B7/BB1 J Exp Med 1993 177 165 173 10.1084/jem.177.1.165 7678111 PMC2190874

[B6] Moreland LW Alten R Bosch F Van den Appelboom T Leon M Emery P Cohen S Luggen M Shergy W Nuamah I Becker JC Costimulatory blockade in patients with rheumatoid arthritis: a pilot, dose-finding, double-blind, placebo-controlled clinical trial evaluating CTLA-4Ig and LEA29Y eighty-five days after the first infusion Arthritis Rheum 2002 46 1470 1479 10.1002/art.10294 12115176

[B7] Kremer JM Westhovens R Leon M Di Giorgio E Alten R Steinfeld S Russell A Dougados M Emery P Nuamah IF Williams GR Becker JC Hagerty DT Moreland LW Treatment of rheumatoid arthritis by selective inhibition of T-cell activation with fusion protein CTLA4Ig N Engl J Med 2003 349 1907 1915 10.1056/NEJMoa035075 14614165

[B8] Kremer JM Dougados M Emery P Durez P Sibilia J Shergy W Steinfeld S Tindall E Becker JC Li T Nuamah IF Aranda R Moreland LW Treatment of rheumatoid arthritis with the selective costimulation modulator abatacept: twelve-month results of a phase iib, double-blind, randomized, placebo-controlled trial Arthritis Rheum 2005 52 2263 2271 10.1002/art.21201 16052582

[B9] Genovese MC Becker JC Schiff M Luggen M Sherrer Y Kremer J Birbara C Box J Natarajan K Nuamah I Li T Aranda R Hagerty DT Dougados M Abatacept for rheumatoid arthritis refractory to tumor necrosis factor alpha inhibition N Engl J Med 2005 353 1114 1123 10.1056/NEJMoa050524 16162882

[B10] Kremer JM Genant HK Moreland LW Russell AS Emery P Abud-Mendoza C Szechinski J Li T Ge Z Becker JC Westhovens R Effects of abatacept in patients with methotrexate-resistant active rheumatoid arthritis: a randomized trial Ann Intern Med 2006 144 865 876 16785475 10.7326/0003-4819-144-12-200606200-00003

[B11] Weinblatt M Combe B Covucci A Aranda R Becker JC Keystone E Safety of the selective costimulation modulator abatacept in rheumatoid arthritis patients receiving background biologic and nonbiologic disease-modifying antirheumatic drugs: A one-year randomized, placebo-controlled study Arthritis Rheum 2006 54 2807 2816 10.1002/art.22070 16947384

[B12] Genovese MC Schiff M Luggen M Becker JC Aranda R Teng J Li T Schmidely N Le Bars M Dougados M Efficacy and safety of the selective co-stimulation modulator abatacept following 2 years of treatment in patients with rheumatoid arthritis and an inadequate response to anti-tumour necrosis factor therapy Ann Rheum Dis 2008 67 547 554 10.1136/ard.2007.074773 17921185

[B13] Simon TA Smitten AL Meng M Franklin J Askling J Lacaille D Wolfe F Suissa S Hochberg M Observed and expected hospitalized infections in the abatacept clinical development program: An updated epidemiological assessment American College of Rheumatology, November; Boston, Massachusetts 2007 FRI 821/849

[B14] Simon TA Covucci A Meng M Skovron ML Descriptive overtime analysis of infections and malignancies in the abatacept clinical development program American College of Rheumatology; November; Boston, Massachusetts 2007 FRI 1039/1271

[B15] Schiff M Keiserman M Codding C Songcharoen S Berman A Nayiager S Saldate C Li T Aranda R Becker JC Lin C Cornet PL Dougados M Efficacy and safety of abatacept or infliximab vs placebo in ATTEST: a phase III, multi-centre, randomised, double-blind, placebo-controlled study in patients with rheumatoid arthritis and an inadequate response to methotrexate Ann Rheum Dis 2008 67 1096 1103 10.1136/ard.2007.080002 18055472 PMC2564802

[B16] Schiff M Pritchard C Zhou X Bahrt K Genovese MC The efficacy of abatacept in patients with active rheumatoid arthritis and an inadequate response to anti-TNF therapy: The ARRIVE trial Arthritis Rheum 2007 56 S391 Abstract 942 10.1136/ard.2008.099218 PMC2756956 19074911

[B17] Symmons DP Silman AJ The Norfolk Arthritis Register (NOAR) Clin Exp Rheumatol 2003 21 S94 99 14969058

[B18] Lacaille D Anis AH Guh DP Esdaile JM Gaps in care for rheumatoid arthritis: a population study Arthritis Rheum 2005 53 241 248 10.1002/art.21077 15818655

[B19] Askling J Fored CM Geborek P Jacobsson LT van Vollenhoven R Feltelius N Lindblad S Klareskog L Swedish registers to examine drug safety and clinical issues in RA Ann Rheum Dis 2006 65 707 712 10.1136/ard.2005.045872 16414975 PMC1798175

[B20] Wolfe F Caplan L Michaud K Treatment for rheumatoid arthritis and the risk of hospitalization for pneumonia: associations with prednisone, disease-modifying antirheumatic drugs, and anti-tumor necrosis factor therapy Arthritis Rheum 2006 54 628 634 10.1002/art.21568 16447241

[B21] DerSimonian R Laird N Meta-analysis in clinical trials Control Clin Trials 1986 7 177 188 10.1016/0197-2456(86)90046-2 3802833

[B22] Wilson EB Hilferty MM The distribution of chi-square Proc Nat Acad Sci 1931 17 684 688 10.1073/pnas.17.12.684 16577411 PMC1076144

[B23] Salliot C Dougados M Gossec L Risk of serious infections during rituximab, abatacept and anakinra treatments for rheumatoid arthritis: meta-analyses of randomised placebo-controlled trials Ann Rheum Dis 2009 68 25 32 10.1136/ard.2007.083188 18203761 PMC2596305

[B24] Dobloug JH Forre O Kvien TK Egeland T Degre M Natural killer (NK) cell activity of peripheral blood, synovial fluid, and synovial tissue lymphocytes from patients with rheumatoid arthritis and juvenile rheumatoid arthritis Ann Rheum Dis 1982 41 490 494 10.1136/ard.41.5.490 6181746 PMC1001029

[B25] Fox RI Fong S Tsoukas C Vaughan JH Characterization of recirculating lymphocytes in rheumatoid arthritis patients: selective deficiency of natural killer cells in thoracic duct lymph J Immunol 1984 132 2883 2887 6609962

[B26] Koetz K Bryl E Spickschen K O'Fallon WM Goronzy JJ Weyand CM T cell homeostasis in patients with rheumatoid arthritis Proc Natl Acad Sci USA 2000 97 9203 9208 10.1073/pnas.97.16.9203 10922071 PMC16846

[B27] Stuck AE Minder CE Frey FJ Risk of infectious complications in patients taking glucocorticosteroids Rev Infect Dis 1989 11 954 963 2690289 10.1093/clinids/11.6.954

[B28] Doran MF Crowson CS Pond GR O'Fallon WM Gabriel SE Predictors of infection in rheumatoid arthritis Arthritis Rheum 2002 46 2294 2300 10.1002/art.10529 12355476

[B29] Lacaille D Guh DP Abrahamowicz M Anis AH Esdaile JM Use of nonbiologic disease-modifying antirheumatic drugs and risk of infection in patients with rheumatoid arthritis Arthritis Rheum 2008 59 1074 1081 10.1002/art.23913 18668604

[B30] Listing J Strangfeld A Kary S Rau R von Hinueber U Stoyanova-Scholz M Gromnica-Ihle E Antoni C Herzer P Kekow J Infections in patients with rheumatoid arthritis treated with biologic agents Arthritis Rheum 2005 52 3403 3412 10.1002/art.21386 16255017

[B31] Curtis JR Xi J Patkar N Xie A Saag KG Martin C Drug-specific and time-dependent risks of bacterial infection among patients with rheumatoid arthritis who were exposed to tumor necrosis factor alpha antagonists Arthritis Rheum 2007 56 4226 4227 10.1002/art.23050 18050253 PMC2561262

[B32] Askling J Dixon W The safety of anti-tumour necrosis factor therapy in rheumatoid arthritis Curr Opin Rheumatol 2008 20 138 144 10.1097/BOR.0b013e3282f4b392 18349742

[B33] Food and Drug Administration. Guidance for Industry. Good Pharmacovigilance Practices and Pharmacoepidemiologic Assessment http://www.fda.gov/cber/gdlns/pharmacovig.htm

[B34] Solomon DH The comparative safety and effectiveness of TNF-alpha antagonists J Manag Care Pharm 2007 13 S7 18 17378700 10.18553/jmcp.2007.13.s1.7 PMC10437747

