# Supplementary material for: Infections requiring hospitalization in the abatacept clinical development program: an epidemiological assessment
Source: Arthritis Res Ther. 2010 Apr 14;12(2):R67. doi: 10.1186/ar2984 (PMC2888222; doi:10.1186/ar2984)
Supplement: Additional file 1 — Abatacept epidemiology study group members. A Word file containing a complete list of all members of the Abatacept Epidemiology Study Group. [file ar2984-S1.DOC]

**Abatacept Epidemiology Study Group Members (Teresa Simon: Chair)**

**Johan Askling and Lars Klareskog,**

Karolinska Institutet, Rheumatology unit d2:01, Karolinska University hospital solna, 171 76 Stockholm, Sweden.

**John's Esdaile and Diane Lacaille**

Arthritis Research Centre of Canada,

895 west 10th ave,

Vancouver,

BC, Canada,

V5Z 1L7

**Jarrod Franklin**

Research Assistant,

arc Epidemiology Unit, School of Medicine

University of Manchester, Manchester, United Kingdom,

**Marc C. Hochberg**Professor of Medicine
Head, Division of Rheumatology & Clinical Immunology
University of Maryland School of Medicine
10 S. Pine St., MSTF 8-34
Baltimore, MD 21201

Prof Alan Silman
Medical Director
Arthritis Research Campaign
Copeman House
St Mary’s Gate
St Mary’s Court
Chesterfield

**Samy Suissa**

James McGill Professor of Epidemiology, Biostatistics and Medicine, McGill University

Director, McGill Pharmacoepidemiology Research Unit Distinguished Scientist, CIHR Royal Victoria Hospital

687 Pine Ave West, R4.29 Montreal, Quebec H3A 1A1 Canada

**Fred Wolfe**

National Data Bank for Rheumatic Diseases
1035 N. Emporia
Suite 288
Wichita, KS 67214
